# Precise Management of n‐Value Distribution at the 2D/3D Interface Enabling Efficient Wide‐Bandgap Perovskite Solar Cells

**DOI:** 10.1002/advs.76791

**Published:** 2026-08-10

**Authors:** Yajie Yang, Jiaxin Ha, Yang Zhang, Yunfan Wang, Ting Jiang, Jingquan Zhang, Lili Wu, Wenwu Wang, Guanggen Zeng, Baoyan Fan, Mohammad Abdul Halim, Xia Hao, Dewei Zhao

**Affiliations:** ^1^ Institute of New Energy and Low‐Carbon Technology & College of Materials Science and Engineering Sichuan University Chengdu China; ^2^ Department of Materials Science and Engineering City University of Hong Kong Hong Kong SAR China; ^3^ Engineering Research Center of Alternative Energy Materials & Devices Ministry of Education Chengdu China; ^4^ College of Materials and New Energy Chongqing University of Science and Technology Chongqing China; ^5^ Department of Materials science and Engineering University of Rajshahi Rajshahi Bangladesh

**Keywords:** 2D/3D perovskite interface, mixed‐dimensional perovskite, perovskite solar cells, phase transition

## Abstract

Introducing a 2D perovskite layer on the surface of 3D perovskite has been broadly recognized as an effective strategy to enhance the performance of perovskite solar cells (PSCs). However, the mechanism governing the 2D phase formation remains uncertain. In this work, the phase transitions of 2D perovskite during spin‐coating and annealing processes has been investigated. Our findings reveal a dimensional phase shift from low to high n‐value 2D phases, driven by the release of organic cations during annealing. Additionally, the spin‐coating process exhibited concentration‐dependent behavior, where higher n‐octylamine hydrobromide (OABr) concentrations predominantly formed n = 1 phases. These observations highlight the complexity of the 2D phase composition at the 2D/3D interface. The coexistence of various 2D phases significantly influences device performance, as a conflict between n = 1 and n ≥ 2 phases was identified. Through forming a well‐balanced proportion of different 2D phases, we achieved a wide‐bandgap perovskite solar cell with a significantly improved power conversion efficiency of 19.29%. This work suggests the critical roles of phase dynamics and n‐value distribution in optimizing 2D/3D interfaces for advancing high‐performance wide‐bandgap PSCs.

## Graphical abstract

This study elucidates the phase transition pathway of surface‐engineered perovskite films. During spin‐coating, n = 1 2D perovskite dominantly forms, while subsequent annealing drives OA^+^ release and induces conversion to higher n‐value 2D phases. The revealed dimensional shift provides new insight into the dynamic evolution of 2D/3D interfaces, providing strategic guidance for the development of stable and efficient wide‐bandgap perovskite solar cells.

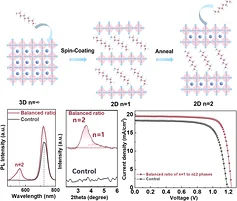

## 1 Introduction

Driven by a rapid enhancement in power conversion efficiency (PCE), which has recently surpassed 27% [1], perovskite solar cells (PSCs) are now regarded as one of the most promising photovoltaic technologies. The tunability of the bandgap [2, 3], ease of fabrication [4], and potential for flexible device applications make this technology a promising candidate for a wide range of applications and commercialization [5, 6] However, defects within the perovskite bulk and at the surface compromise device performance and the long‐term stability [7, 8]. To address these challenges, various passivators have been used for surface treatment. This process usually forms a new passivation layer on the perovskite surface, which not only reduces defects, suppresses charge recombination, improves power output, but also enhances stability by preventing degradation [9, 10].

Given the significant role of surface treatment, surface passivation techniques have been extensively developed. Lewis acid [11], Lewis base [12], polymeric layers [13], and small molecule self‐assembly layers [14] have been proven effective as surface treatment agents. Among them, 2D perovskite layer passivation has gained significant attention due to its ability to passivate defects, moderate energy level alignment, and serve as a barrier against water and oxygen infiltration [15, 16, 17].

When bulky organic cations are introduced to the surface of the perovskite, they tend to interact with the A‐site cations in the crystal structure of 3D perovskite. This interaction can result in the formation of a molecular layer on top of the 3D perovskite layer. In some cases, organic cations may replace the A‐site cations, disrupt the crystal lattice and cause the separation of octahedral BX_6_ slabs. If the structure contains only one single BX_6_ slab, it is classified as a 2D perovskite (n = 1). When multiple BX_6_ layers are present, the structure is referred to as quasi‐2D perovskite, with the n‐value corresponding to the number of slabs [18]. Then, 3D perovskite can be considered as a quasi‐2D perovskite with an infinite n‐value. The newly formed 2D structure on the perovskite surface contributes to the development of a 2D/3D heterostructure.

Post‐treatment is commonly processed to generate 2D/3D heterostructure [19, 20, 21, 22]. The formation mechanism of 2D perovskite phase has been investigated to gain insights for further enhancing device efficiency and stability [23, 24]. Proppe et al. [25] observed a dimensional shift from n = 3→2→1 after introducing vinylbenzylammonium (VBA) ligand cations on 3D perovskite surface. Kodalle et al. [26] reported the film evolution by dripping phenylethylammonium (PEAI) on 3D perovskite. They observed that the n = 1 Ruddlesden‐Popper (RP)‐phase appeared before n > 1 RP‐phase, indicating that the 2D perovskite phase shifts to higher n‐value phases in the PEAI‐treated film. Ramakrishnan et al. [27] investigated the dimensional shift during spin‐coating. They dissolved 3‐aminomethylpyridinium diiodide (3‐AMPY) in isopropanol (IPA) and 2, 2, 2‐trifluoroethanol (TFE) respectively (both of this solvent has 0.5 vol% DMF addition), and compared the 2D perovskite formation process. After IPA treatment, a PbI_2_‐rich film was obtained, which gives the ligand molecule opportunities to react with PbI_2_ and form low n‐value first. In contrast, TFE preserves the 3D surface, initially generating high n‐value and ultimately presenting a n‐value change from high to low. Guo et al. [28] also compared 2D perovskite formation with different ligand cations, finding that the formation of low‐n phases is influenced by the chain length and steric hindrance of the alkyl terminals of the spacer cations. Tan and coworkers [29] reported that the incorporation of methylammonium (MA) additives in mixed solvents (e.g., dimethyl sulfoxide/isopropanol) enables precise control over the crystallization kinetics of 2D perovskites (n = 2). The resulting 2D/3D heterostructure devices delivered a PCE of 25.9%.

During the spin‐coating process, two pathways for n‐value conversion were identified: (1) the formation of the n = 1 phase from cation + PbI_2_, followed by a transition from lower n‐values to higher n‐values; (2) the formation of cation + 3D perovskite, subsequently leading to a transition from higher n‐values to lower n‐values. However, the driving factors behind these mechanisms have not been clearly analyzed yet. A more in‐depth understanding of the phase transition and their driving factors is highly required.

In this work, we choose a 1.72 eV wide‐bandgap perovskite (FA_0.8_Cs_0.2_Pb(I_0.7_Br_0.3_)_3_) to investigate the evolution of the 2D perovskite structure formed by n‐octylamine (OA^+^) during spin‐coating and annealing. This bandgap is relevant for tandem top cells, and this composition is widely used as a model system, ensuring that our mechanistic findings are generalizable across wide‐bandgap perovskites. During annealing, we observed a transition from low to high n‐values in 2D perovskites. X‐ray photoelectron spectroscopy (XPS) and Time‐of‐flight secondary ion mass spectrometry (TOF‐SIMs) analysis revealed a sharp decrease in OA^+^ concentration at the surface, with no evidence of diffusion into the perovskite bulk. This suggests that the escape of OA^+^ from the surface drives the n‐value transition. We also identified a concentration‐driven phenomenon in 2D phase formation: higher OA^+^ concentrations favor n = 1 phases, while lower concentrations favor n ≥ 2 phases. These phases undergo dimensional increase during annealing. Lastly, we found that the n = 1 phase better passivates defects, while the n ≥ 2 phase enhances charge transport. Thus, a balanced ratio of n = 1 to n ≥ 2 phases is substantial in achieving optimal PSC performance. This work provides insights into 2D phase evolution and their role in passivation strategies to enhance PSC performance.

## 2 Results and Discussion

### 2.1 Mechanism Insights during Annealing

To better comprehend the mechanism of 2D perovskite formation, we introduced n‐octylammonium bromide (OABr) on the perovskite surface to form the 2D/3D heterostructure. X‐ray diffraction (XRD) was initially conducted on perovskite films before (denoted as OABr‐NA) and after annealing process (denoted as OABr‐A) to investigate the crystal structure. Figure 1a illustrates that after spin‐coating 0.5 mg/mL OABr precursor onto the surface of perovskite layer, a characteristic diffraction peak emerges at 3.2°. Following the annealing process, this peak shifts to 3.3° with an increase in intensity. To determine the phase corresponding to this newly formed peak, we fabricated 2D perovskites with different n values, as presented in Figure S1. By comparison, the characteristic peak at 3.14° in the n = 3 2D perovskite closely matches the peak observed in Figure 1a. However, the peak position of the n = 3 phase in the 2D/3D heterostructure is slightly higher than that of the pure n = 3 2D perovskite. The slight shift of the n = 3 diffraction peak from 3.14° in the pure 2D film to ∼3.2–3.3° in the 2D/3D heterostructure is attributed to compressive strain induced by the lattice mismatch at the 2D/3D interface, which reduces the out‐of‐plane d‐spacing of the 2D layer [30, 31]. The similar trend is evident in Figure S1, where the 2D characteristic peaks exhibit a shift toward lower angles as the n‐value increases. This suggests that the interplanar spacing along the c‐axis in 2D perovskite, perpendicular to the substrate, increases with the n‐value, as reflected in the lower‐angle shift of the characteristic peaks [32].

**FIGURE 1:**
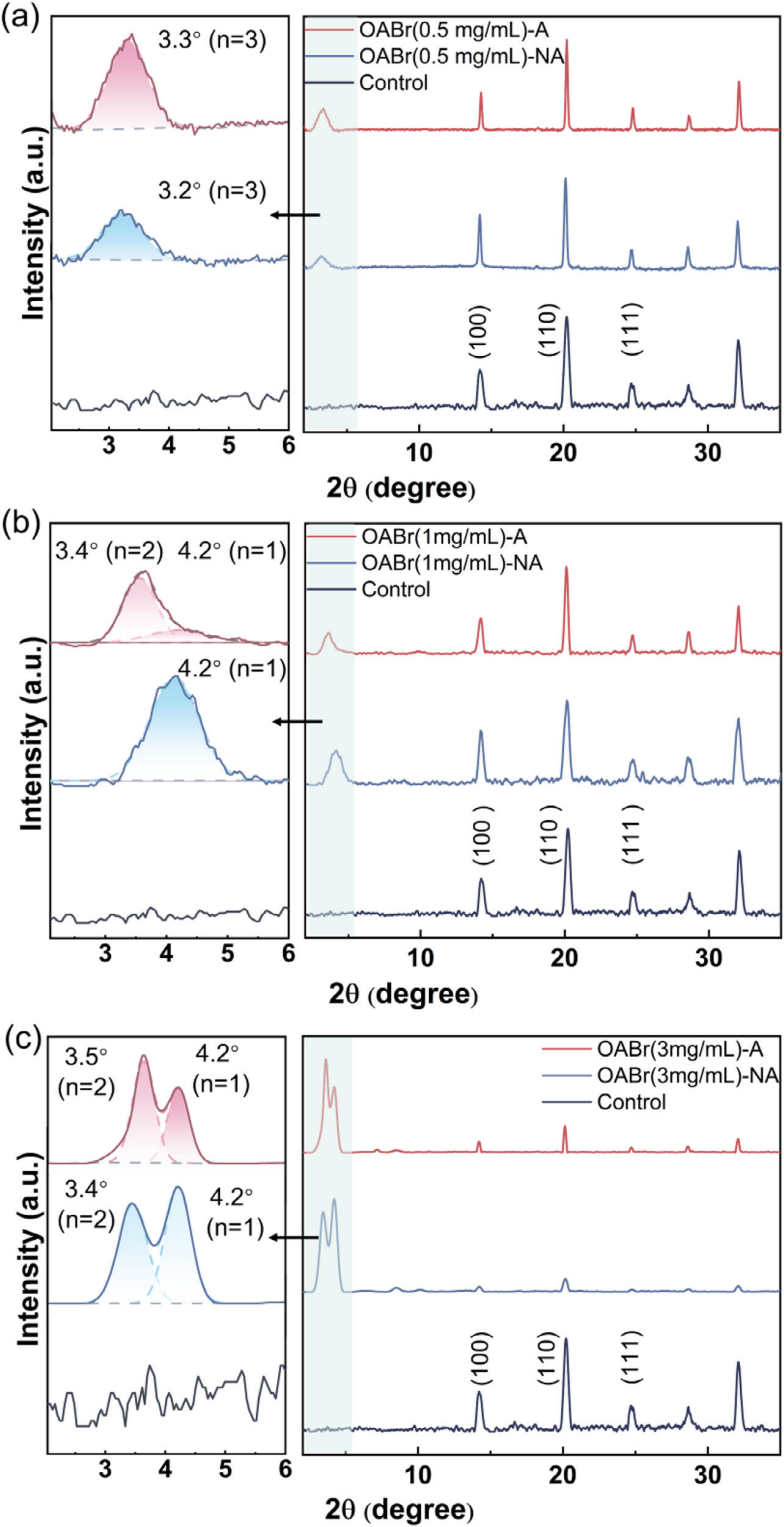
XRD patterns of (a) 0.5 mg/mL, (b) 1 mg/mL, (c) 3 mg/mL OABr treated films before (marked as NA) and after annealing (marked as A) process.

When the OABr precursor concentration increases to 1 mg/mL, a new characteristic peak appears at 4.2°, as depicted in Figure 1b. Considering both experimental and documented reports [33], the peak at 4.2° is attributed to the n = 1 2D phase. After the annealing process, the peak intensity of the n = 1 2D phase decreases, while a new characteristic peak emerges at 3.4°, which can be attributed to the n = 2 2D phase (appearing at 3.4° in Figure S1). Through diffraction peak analysis, the annealed perovskite film (OABr (1 mg/mL)‐A) is found to exhibit a phase proportion of 2D(n = 1):2D(n = 2) = 1:3. This indicates a phase transition from the n = 1 to the n = 2 2D phase during annealing. When the precursor concentration is increased to 3 mg/mL, the n = 1 and n = 2 2D phases are simultaneously detected in the as‐deposited film (Figure 1c). After annealing, a similar trend is observed, where the n = 1 phase weakens while the n = 2 phase intensifies. Before annealing, the film has a 2D_(n = 1)_:2D_(n = 2)_ ratio of 51:49, whereas after annealing, this ratio changes to 42:58.

We then performed XPS measurements to further investigate the factors driving the 2D phase transition. Figure 2a,b display the characteristic peaks corresponding to C 1s and N 1s. In the N 1s spectra, the C─N/C═N peaks show a slight shift to lower binding energy upon the addition of OABr, from 400.77 eV (Control) to 400.37 eV (OABr‐NA and OABr‐A). This shift supports the strong chemical interaction between OABr and the perovskite. Furthermore, a new NH_3_^+^ peak appears in both OABr‐NA and OABr‐A films, indicating that the OA^+^ is present within approximately 10 nm of the films surface. Additionally, the weighting factor of NH_3_^+^ decreases after annealing process, suggesting the loss of OA^+^ on the perovskite surface [34, 35]. A similar peak shift and OA^+^ loss phenomenon can also be observed in the C 1s spectrum. Moreover, as shown in Figure S2, the binding energy of Pb 4f_5/2_ (138.57 eV) and Pb 4f_7/2_ (143.37 eV) of control film shifts to 138.27 and 143.07 eV, respectively, after OABr treatment. The decrease in binding energy can be attributed to the introduction of OABr that modifies the chemical environment around the Pb atoms on the film surface, leading to a reorganization of the electron cloud. In particular, the unpaired electrons provided in OA^+^ donate electrons to Pb^2+^ empty orbits, which results in a shift of the binding energy toward lower values [36].

**FIGURE 2:**
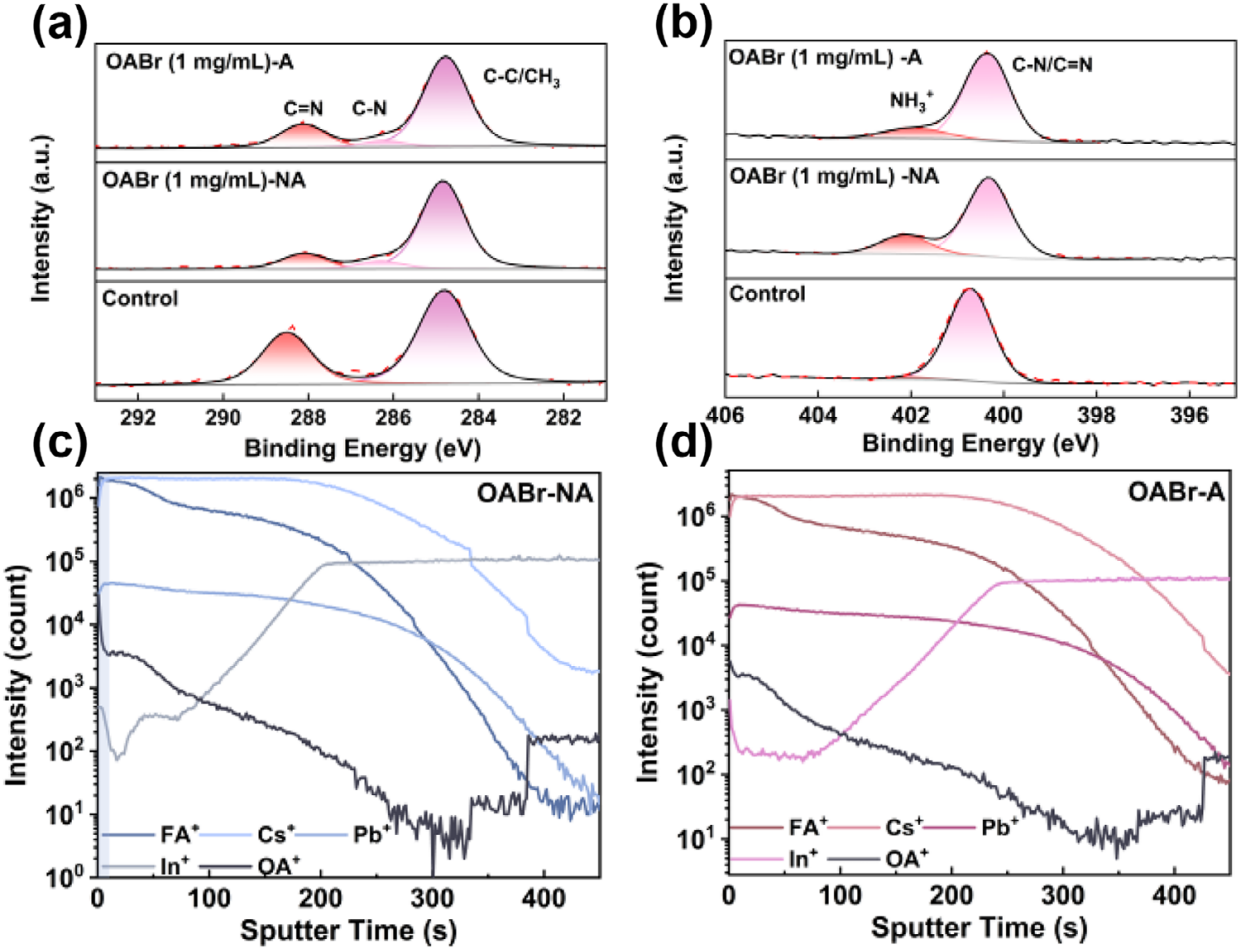
XPS patterns of OABr treated films before and after annealing process: (a) C 1s, (b) N 1s, and TOF‐SIMs results of the (c) OABr‐NA and (d) OABr‐A films surface.

TOF‐SIMs results confirm the loss of OA^+^ from the perovskite surface during annealing process. As illustrated in Figure 2c,d and Figure S3, the C_8_H_20_N^+^ (OA^+^) exhibits an 80% decrease in quantity on the perovskite film surface before and after annealing. Moreover, a significant decrease in the OA^+^ content is observed within the first 9 s of sputtering. Considering the SIMS sputtering rate calibrated on PMMA (∼0.8 nm/s), the first ∼9 s of measurement corresponds to a depth of about 7.2 nm, within ∼10 nm from the film surface. We therefore infer that the drastic change in OA^+^ content occurs in this near‐surface region, consistent with the XPS results. Notably, no increase in OA^+^ content is observed at depths greater than 10 nm before and after annealing, indicating the absence of cation migration. Therefore, we propose that the phase transition of the OA^+^‐induced 2D/3D structure primarily occurs in the front surface region of the perovskite film during annealing [35]. Furthermore, the loss of OA^+^ from the surface during heating (rather than diffusion into deeper layers of the film) facilitates the transformation from the n = 1 to the n = 2 2D phase. This observation aligns with the differences in cation content between the different 2D phases.

### 2.2 Concentration‐Driven Phase Formation during Spin‐Coating

To gain insights into the mechanism governing 2D/3D interface formation, we analyzed the impact of OABr concentration on the development of 2D phases. The XRD results of the films before and after annealing are shown in Figure 1. A characteristic peak at 3.2° (n = 3) was observed after spin‐coating with 0.5 mg/mL OABr solution. For the OABr (1 mg/mL)‐NA group, a distinct 2D‐phase peak corresponding to n = 1 appeared at 4.2°, while in the OABr (3 mg/mL)‐NA group, additional peaks at 4.2° (n = 1) and 3.6° (n = 2) emerged. We propose that, at low concentrations, the spin‐coating process tends to form high n‐value 2D layers on the 3D perovskite surface. When the OABr concentration increases to 1 mg/mL, an n = 1 2D phase is formed. In samples with higher concentrations, both n = 1 and n = 2 phases coexist.

In parallel, we performed photoluminescence (PL) measurements to verify the phase formation during spin‐coating. As shown in Figure 3, a comparison is made between films with different concentrations of OABr. With increasing OABr concentration, the intensity of the 2D‐phase PL peak within the range of 510–560 nm increases, accompanied by noticeable changes in the peak shape. To further analyze this phenomenon, peak deconvolution was performed on the 2D PL peaks of films treated with various OABr concentrations. The detailed deconvolution of the 2D PL peaks, including the raw data, individual fitted peaks (n = 1 and n = 2), cumulative fit, baseline, and the corresponding fitting parameters (peak center, FWHM, peak area, and adjusted R^2^), is provided in Figure S4 for all treated groups. This analysis identified the characteristic n = 1 2D peak at 510 nm and the n = 2 2D peak at 560 nm [37, 38, 39]. The absence of characteristic peaks for higher n‐value 2D phases in the spectra is attributed to their inherently high exciton binding energy, which makes them difficult to be detected at room temperature [40]. In the low‐concentration OABr‐treated group (1 mg/mL), the n = 1 2D phase is absent immediately after spin‐coating (Figure 3a), whereas in the high‐concentration OABr‐treated group, the n = 1 2D phase is observed after post spin‐coating (Figure 3b,c). The PL results are consistent with the previously discussed concentration‐driven formation of 2D phases during the spin‐coating process. Furthermore, the n = 2 phase peak becomes more intense relative to the n = 1 phase, upon annealing. This observation indirectly supports the transition of 2D phases from lower n‐values to higher n‐values during the annealing process. It is worth noting that although XRD patterns indicate the presence of highern (n ≥ 3) 2D perovskite phases, their corresponding PL peaks are not observed at room temperature. This can be attributed to the lower exciton binding energy of highern phases, resulting in stronger thermal quenching and consequently no observable PL emission at room temperature [41].

**FIGURE 3:**
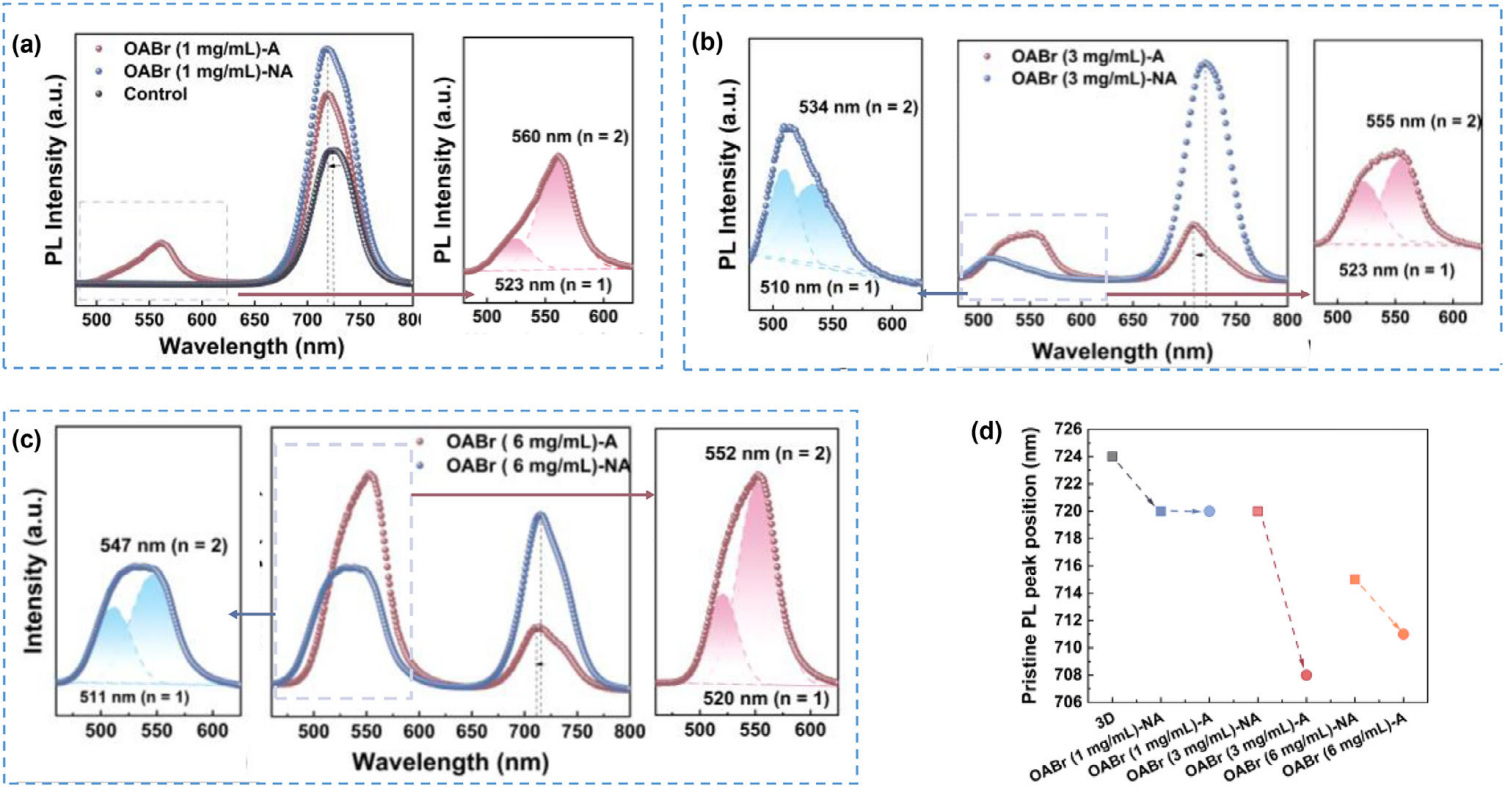
Steady‐state PL spectra. (a) 1 mg/mL OABr treatment, (b) 3 mg/mL OABr treatment and (c) 6 mg/mL OABr treatment before and after annealing process; (d) Pristine perovskite PL peak position.

The concentration‐dependent phase formation process can be driven by multiple factors. Initially, no characteristic peaks of unreacted OABr were observed on the surface of the spin‐coated films. This indicates that the formation of 2D phases with OABr as a passivating agent occurs spontaneously at room temperature. Consequently, different 2D phases may form on the surface of the 3D perovskite after the spin‐coating process. However, the formation energy associated with the n = 2 phase is lower than that of the n = 1 phase. During the formation process, the hydrogen bonding between the OA^+^ cations and the perovskite, along with the stronger van der Waals interactions in the n = 1 phase [42], makes the evolution of the n = 1 phase more energetically demanding. As a result, the n = 1 phase is more likely to appear as a distinct diffraction peak only in samples with higher concentrations. At higher concentrations, a greater proportion of molecules possess energies above the thermodynamic average, which enables more molecules to acquire the energy necessary for n = 1 phase formation. This increase in available reactive ions leads to the development of the n = 1 phase only at higher concentrations [43]. However, this does not fully account for the observation that at higher concentrations, the n = 2 phase coexists with and even exceeds the n = 1 phase. We propose that the formation of 2D perovskites follows a sequential nucleation pathway governed by local concentration gradients. Once the n = 1 phase nucleates and consumes a portion of the organic cations in a localized region, the effective OABr concentration in the remaining precursor drops below the critical threshold required for further n = 1 formation. Under these conditions, the system instead favors the nucleation of highern phases such as n = 2, which have a lower formation energy barrier. This sequential process rationalizes the coexistence of n = 1 and n = 2 at high concentrations, with n = 2 being the dominant component [44].

Additionally, the PL peaks of 3D perovskite were found to shift slightly to higher energy as the OABr concentration increased (Figure 3d). This shift is likely due to the incorporation of Br^−^, which contributes to an increase in the bandgap of the wide‐bandgap perovskite and, consequently, results in the observed blue shift. This phenomenon can be explained by the influence of the Br/I ratio at the X‐site on the bandgap of the wide‐bandgap perovskite. Specifically, the incorporation of Br^−^ ions from OABr into the film induces a lattice contraction due to the smaller ionic radius of Br^−^ compared to that of I^−^. When Br^−^ ions enter the BX_6_ octahedral framework, this contraction leads to structural changes [45, 46]. The conduction band minimum (CBM) and valence band maximum (VBM) are primarily determined by the orbitals of the B‐site and X‐site ions. As the X‐site ion is modified, the bandgap exhibits an almost linear change with the average ionic radius of the X‐site ions [47]. Consequently, the introduction of Br^−^ results in an increase in the bandgap of the 3D perovskite. In contrast, in Figure S5, where OAI was used as a passivator, a redshift in the 3D PL peak was observed, indicating a decrease in the bandgap upon the introduction of I^−^. Meanwhile, the continued blue shift of the 3D perovskite PL peak after annealing can be considered the further diffusion of Br^−^ ions during the annealing process. Furthermore, in all OABr‐treated groups at various concentrations, the intensity of the 3D perovskite PL peak significantly decreased after annealing. This decrease is likely due to differences in carrier transport and defect passivation capabilities between different 2D phase [9].

Overall, we observe a concentration‐driven n‐value formation process during the spin‐coating stage, which governs the formation of different n‐value 2D phases at the 2D/3D heterojunction interface. During annealing, a transition toward higher n‐value 2D phases occurs due to the detachment of larger organic cations. The interplay of these two formation stages collectively influences the n‐value distribution of 2D phases at the 2D/3D heterojunction interface, providing a foundation for precise management over its 2D phase distribution.

### 2.3 Interface Carrier Dynamics

As mentioned above, OABr (1 mg/mL) group is the key concentration for the appearance of n = 1 2D phase after spin‐coating and relatively simple 2D phases remaining after annealing. Therefore, we fixed the OABr concentration at 1 mg/mL to investigate the surface morphology and potential of OABr‐treated films and further understand the influence of shifted n‐value. The surface (Figure 4a–c) and cross‐sectional SEM images (Figure S6) illustrate the particle size of perovskite films and the grain size distribution is provided in Figure 4d,e. The average grain size of control and OABr‐NA films is 277 nm and 269 nm respectively, while after annealing, the average grain size increases to 301 nm. This indicates the recrystallization process during annealing, align with the 2D phase shift behavior. Figures S7 presents the AFM images, revealing that film with annealing process has a smaller root‐mean‐square roughness (RMS) than that of control and OABr‐NA films. The smoother surface can contribute to the contact between perovskite and hole transport layer (HTL), leading to better photovoltaic performance [48].

**FIGURE 4:**
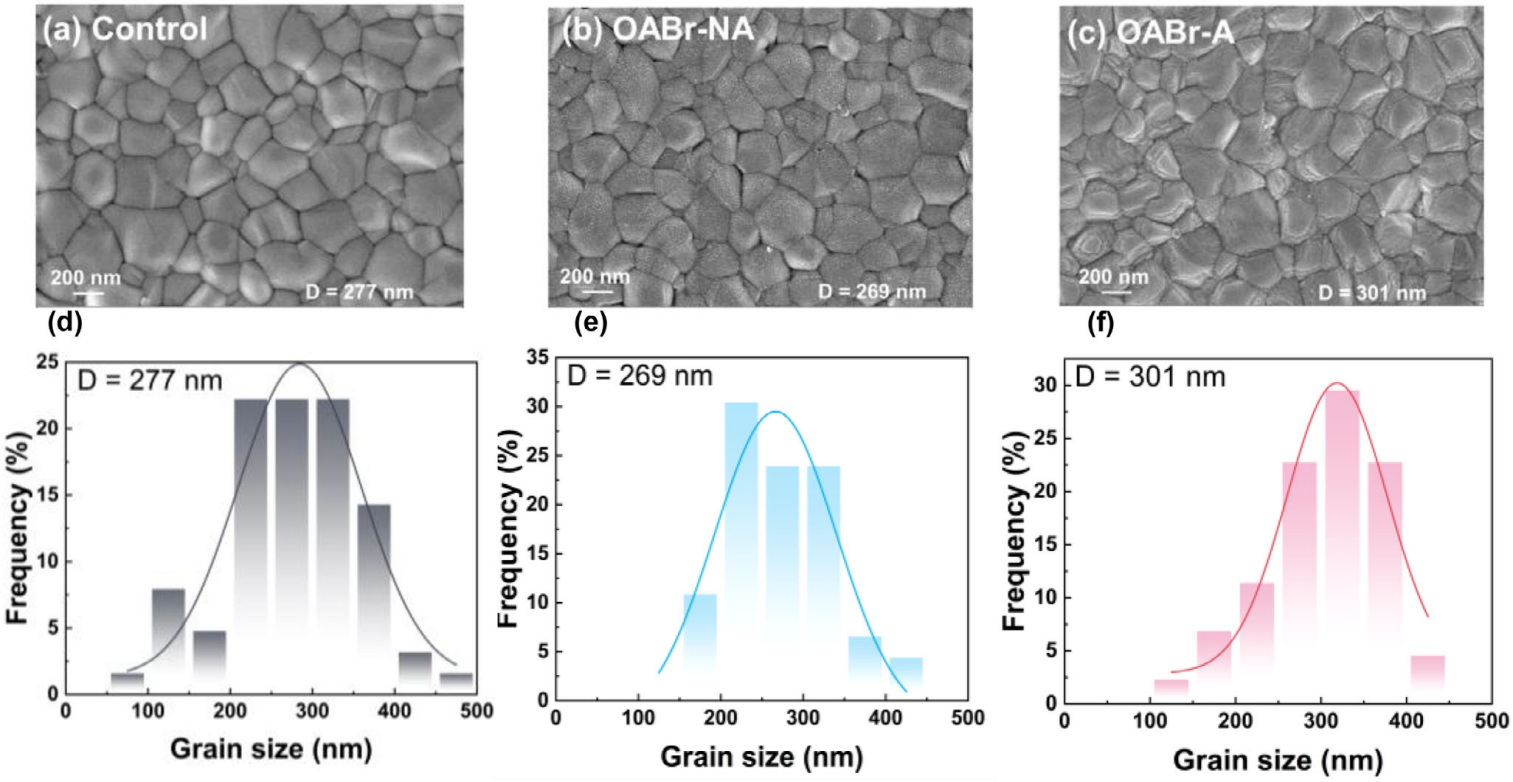
SEM results of perovskite films. (a) Surface SEM images of the control group, (b) OABr‐NA, and (c) OABr‐A perovskite films, along with grain size distribution statistics of (d) the control group, (e) OABr‐NA, and (f) OABr‐A films.

To further evaluate the energy level alignment of OABr‐treated films before and after annealing, we performed ultraviolet photoelectron spectroscopy (UPS) measurement. As shown in Figure 5a, the work functions of these films are 3.92, 3.79 and 3.75 eV corresponding to control, OABr‐NA and OABr‐A films, respectively. Moreover, the distance between VBM and Fermi level (*E*_F_) were determined from the Fermi onset region in Figure 5b, which are calculated to be 1.65, 1.68 and 1.69 eV, respectively. In addition, the bandgaps (*E*_g_) of these films are calculated to be 1.72 eV as depicted in Figures S8 and S9. The energy‐level diagram in Figure 5c reveals a progressive improvement in band alignment upon OABr treatment. Quantitatively, the conduction band offset (CBO) and valence band offset (VBO) between the perovskite and Spiro‐OMeTAD decrease from 0.65 and 0.37 eV (Control) to 0.52 and 0.24 eV (OABr‐A), respectively. The smallest VBO of OABr‐A indicates a reduced hole injection barrier. Consequently, the VBM of OABr‐A is aligned more closely with the HOMO of Spiro‐OMeTAD, leading to superior energy matching and facilitated carrier extraction, which is consistent with the improved fill factor (FF) and short‐circuit current density (*J*_SC_)_observed in OABr‐A devices [49].

**FIGURE 5:**
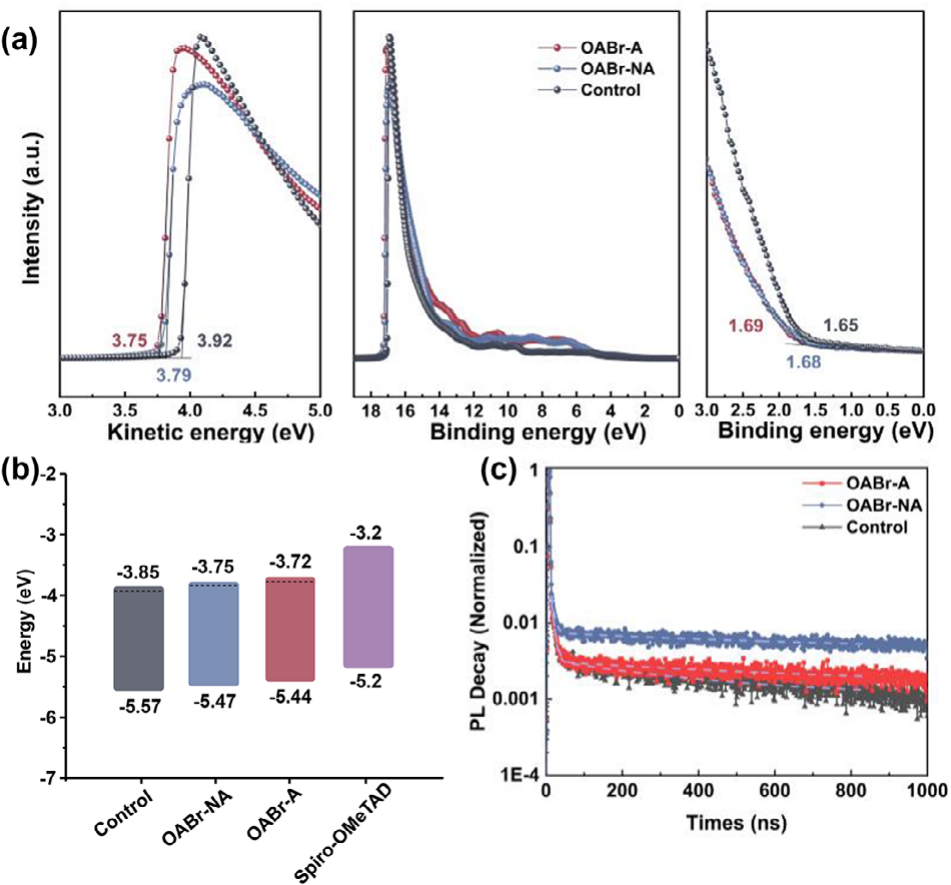
(a) UPS spectra of the control and OABr‐NA and OABr‐A perovskite films; (b) energy band diagram of perovskite films and Spiro‐OMeTAD. (c) Time‐resolved photoluminescence (TRPL) spectra of the control and OABr‐NA and OABr‐A perovskite samples.

TRPL measurement was performed to estimate the carrier lifetime (Figure 5c). The TRPL curves were fitted and calculated according to equation (1) [50]:

(1) $$Y=A_{1}\exp\left(\frac{-t}{\tau_{1}}\right)+A_{2}\exp\left(\frac{-t}{\tau_{2}}\right)+\gamma_{0}$$

where γ_0_ denotes the decay constant, A_1_ and A_2_ denote the decay amplitude. The τ_1_ and τ_2_ of OABr‐A film are 8.10 and 787.27 ns, while those of OABr‐NA film are 7.13 and 706.07 ns, respectively. All of these values are higher than those of 6.75 and 368.30 ns for the control group. The OABr‐A film demonstrates a higher carrier lifetime compared to the OABr‐NA. Thus, the enhanced ability of the 2D phase components on the OABr‐A film to effectively suppress non‐radiative recombination [51].

### 2.4 Performance of PSCs

Clearly, the n = 2 2D phase forms after annealing and exhibits improved energy level alignment. In contrast, the n = 1 2D phase forms prior to annealing and demonstrates PL intensity, indicating enhanced defect passivation capability. Therefore, by regulating the concentration‐driven phase formation during the spin‐coating and the dimensional transition during annealing, the composition of the resulting 2D phases can be precisely tailored, enabling systematic optimization of device performance.

In this study, the devices were designed with a structure of ITO/SnO_2_/(FA_0.8_Cs_0.2_)Pb(I_0.7_Br_0.3_)_3_/OABr/Spiro‐OMeTAD/Au and compared the performance of PSCs with different OABr concentrations after annealing. The current density‐voltage (*J*‐*V*) curves of the OABr‐A and control champion devices were obtained under AM1.5G simulated sunlight and depicted in Figure 6a.

**FIGURE 6:**
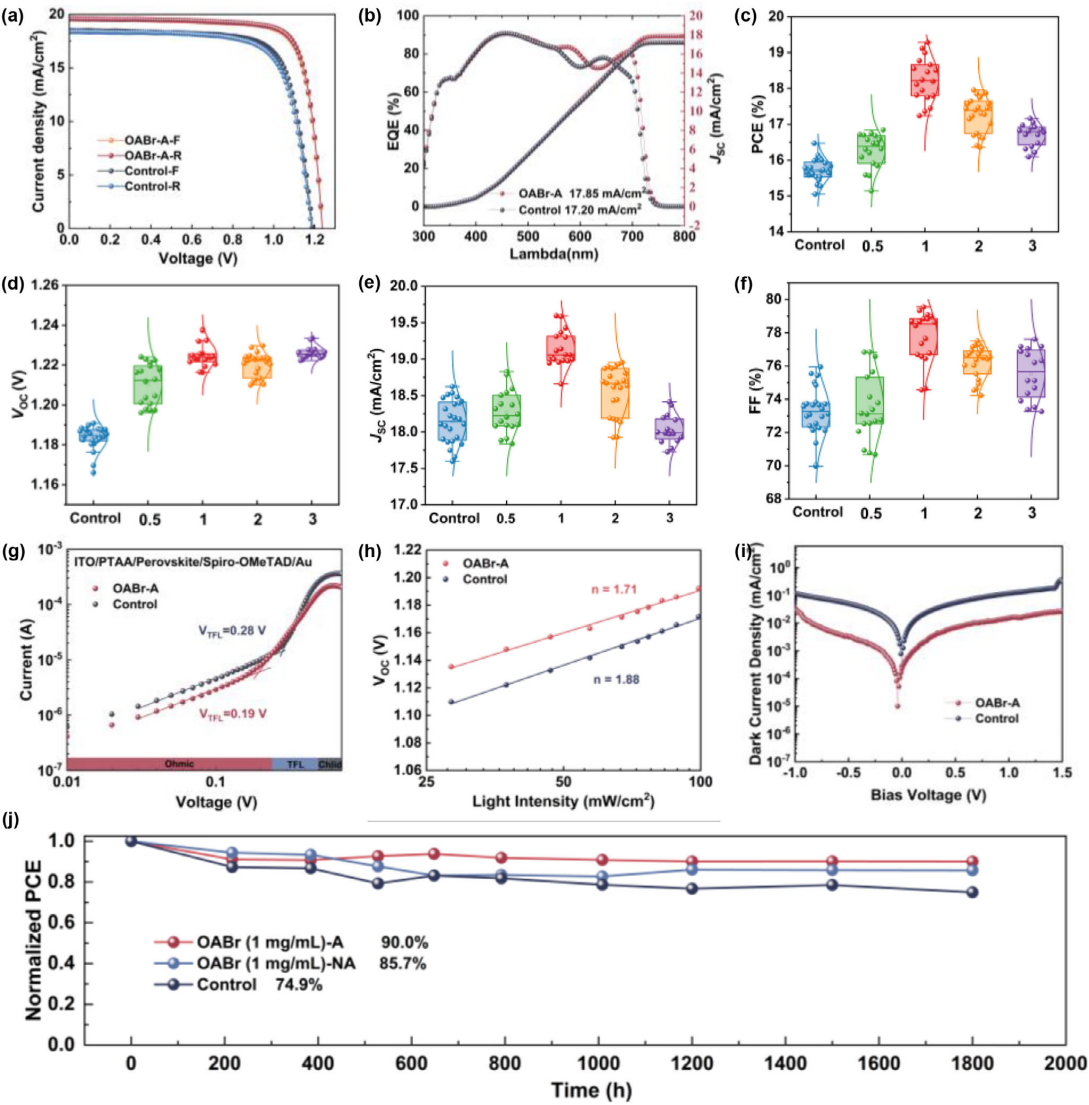
(a) *J−V* curves of the OABr‐A and control champion devices. (b) EQE measurements of the champion devices. Statistics of photovoltaic parameters for the PSCs, (c) PCE, (d) *V*_OC_, (e) FF, and (f) *J*_SC_ with 0 mg/mL (control), 0.5L, 1, 2, and 3 mg/mL OABr treatment (with annealing). The number of tested devices (n) for each group: 24 for control (0 mg/mL) and 2 mg/mL; 18 for 0.5, 1, and 3 mg/mL. (g) SCLC curves for hole‐only devices. (h) The light‐intensity dependent *V*_OC_ of PSCs. (i) Dark *J–V* tests. (j) Stability measurement of the unencapsulated devices aged in dry air (<10% RH) in the dark, with periodic *J–V* measurements under simulated sunlight (AM 1.5G, 100 mW cm^−2^, calibrated with a standard silicon reference cell) at each indicated time point.

The champion device appeared in the 1 mg/mL group exhibiting a PCE of 19.29%, an open‐circuit voltage (*V*_OC_) of 1.24 V, a *J*_SC_ of 19.58 mA/cm^2^ and a FF of 79.55 %. In contrast, the champion device of the control group achieved a PCE of 16.47%, a *V*_OC_ of 1.19 V, a *J*_SC_ of 18.47 mA/cm^2^ and an FF of 74.85 %. The parameters of reverse scan (R) and forward scan (F) can be seen in Table S1. As discussed in the XRD results above, the OABr‐A (1 mg/mL) group is the turning point for most of the n = 1 phases to be converted to n = 2 phases, resulting in an optimal ratio of 2D phase (2D_(n = 1)_:2D_(n = 2)_ = 1:3) to achieve the champion device. This demonstrates that precisely regulating the proportion of 2D phases in the 2D/3D heterojunction can enable the optimization of device performance as expected. The optimal device group not only benefits from the enhanced *V*_OC_ but also maintains high *J*_SC_ and FF, owing to the superior ratio of the n = 1 and n ≥ 2 phases on the film surface. Figure 6b shows the external quantum efficiency (EQE) curves for Control and OABr (1 mg/mL)‐A devices. the integrated *J*_SC_ are 17.20 and 17.85 mA/cm^2^, respectively, in agreement with the *J*_SC_ values derived from the *J–V* curves.

To further elucidate the impact of different 2D phases on device performance, we compared the PCE, *V*_OC_, *J*_SC_, and FF across various groups (Figure 6c–f) and the mean ± SD for PCE, *V*_OC_, *J*_SC_, and FF are shown in Table S2. When the OABr concentration was < 1 mg/mL, the perovskite surface was dominated by n ≥ 2 2D phases. At concentrations ≥ 1 mg/mL, the n = 1 2D phase began to form and exert its influence.

In n ≥ 2 phases dominated region, both FF and *J*_SC_ increased steadily with concentration, reaching their maximum at 1 mg/mL. In the n = 1 phase dominated region, both FF and *J*_SC_ declined. In the case of *V*_OC_, it improved in the n ≥ 2 phases dominated region and showed a further increase once the n = 1 phase was present. This indicates that the n = 1 phase is more effective in reducing non‐radiative recombination by passivating surface defects, thereby boosting *V*_OC_ [52]. The accompanying drop in *J*_SC_ and FF in the n = 1 phase dominated region can be attributed to its more insulating nature, which hinders carrier transport. As for the n ≥ 2 2D phases, they lack the same defect‐passivation ability, as reflected by the modest *V*_OC_ improvement in that regime. However, their lower dielectric confinement compared to the n = 1 phase enables stronger carrier separation and transport [24], which benefits FF and *J*_SC_.

A competitive relationship exists between the n = 1 phase and the n ≥ 2 phases. While the n = 1 phase enhances *V*_OC_ through excellent defect passivation, its insulating nature limits carrier transport. Conversely, n ≥ 2 phases facilitate carrier transport but provide weaker defect passivation.

The space‐charge‐limited‐current (SCLC) method were performed to identify the defect density. The hole‐only device structure in this measurement is Glass/ITO/PTAA/ (FA_0.8_Cs_0.2_)Pb(I_0.7_Br_0.3_)_3_/OABr/Spiro‐OMeTAD/Au. As observed in Figure 6g, the trap‐filled limit voltages (*V*_TFL_) of control and OABr (1 mg/mL)‐A films are 0.28 V and 0.19 V, respectively. The trap density (*N*_d_) can be calculated via Mott−Gurney equation (2) [53]:

(2) $$N_{d}=\frac{2\varepsilon \varepsilon_{0}V_{TFL}}{eL^{2}}\;$$

where ε is the relative permittivity, ε_0_ is the vacuum permittivity, *L* is the thickness of the perovskite film. The *N*_d_ of control film is 3.43 × 10^15^ cm^−3^, while that of OABr (1 mg/mL)‐A film is 2.33 × 10^15^ cm^−3^. The reduced *N*_d_ indicates that the mixed 2D phase in OABr‐treated film effectively passivated the defects and thereby suppressing the non‐radiative recombination. To scrutinize the carrier recombination mechanism in device, the light intensity dependence of *V*_OC_ was conducted as presenting in Figure 6h. The correlation between light intensities and *V*_OC_ can be expressed as equation 3 [54]:

(3) $$V_{OC}=\frac{n_{id}K_{B}T}{e}\ln\left(\frac{I}{I_{0}}+1\right)\;$$

where *K*_B_ is the Boltzmann constant, *T* is the Kelvin temperature, e is the elementary charge, *I* represent the light intensity, *I*_0_ is the initial light intensity under AM 1.5G simulated light and *n*_id_ is the diode ideal factor. The *n*_id_ of OABr (1 mg/mL)‐A device is 1.71, which is closer to 1 compared to that of Control device (*n*_id_ = 1.88). This result indicating that the trap density in the perovskite significantly decreases following treatment with an optimized concentration of OABr. To further validate the role of mixed 2D phase in non‐radiative recombination in the OABr (1 mg/mL)‐A, the dark *J‐V* tests were conducted (Figure 6i). The OABr‐NA films exhibit lower dark current compared to the control group, with the OABr‐A films showing the lowest dark current.

As shown in Figure 6j, devices were stored in dark environment in dry air at 25°C to test storage stability. The unpackaged OABr‐A device demonstrated superior storage stability, maintaining 90% of its initial efficiency after 1800 h of storage, higher than that of OABr‐NA device (85.7%) and Control device (74.9%). The improved stability of OABr‐A devices is attributed to the outstanding stability of n ≥ 2 2D phase [55, 56]. Additionally, the reduced trap density in perovskite also mitigates ion migration, thereby suppressing the phase segregation commonly observed in wide‐bandgap perovskites. This enhancement of phase stability subsequently contributes to the improved environmental stability of the device [57, 58].

## 3 Conclusion

We demonstrated that introducing OABr on the surface of 3D perovskites induces a dimensional transition of 2D perovskites from low to high n values during annealing, driven by the release of bulky organic cations. In contrast, spin‐coating favors the formation of the n = 1 phase at high OABr concentrations. The coexistence of n = 1 and higher‐order (n ≥ 2) phases introduces a competition between effective defect passivation and efficient charge transport. By optimizing the OABr concentration, we achieved a champion wide‐bandgap perovskite solar cell with a PCE of 19.29%. This work clarifies the role of distinct 2D phases in surface passivation and guides the rational selection of passivators for high‐performance devices.

## 4 Experimental Section

### 4.1 Materials

Formamidinium iodide (FAI) was supplied by Greatcell Solar Materials Pty Ltd; Lead iodide (PbI_2_), n‐octylamine Hydrobromide (OABr), n‐octylammonium iodide (OAI), lead iodide (PbI_2_), cesium iodide (CsI) were purchased from TCI; Chlorobenzene (CB), dimethylformamide (DMF), dimethyl sulfoxide (DMSO), isopropanol (IPA) were purchased from J&K Scientific Ltd. Lead bromide (PbBr_2_), bis(trifluoromethane)sulfonimide lithium salt (Li‐TFSI), 4‐tert‐butylpyridine (tBP) and spiro‐OMeTAD were purchased from Xi'an Polymer Light Technology Corp. ITO glasses were purchased from Suzhou Shang Yang Solar Technology Company. FK209 Co (III) TFSI was obtained from Luminescence technology. SnO_2_ colloid precursor (15% in H_2_O colloidal dispersion) was purchased from Alfa Aesar.

### 4.2 Perovskite Precursor Preparation

To prepare 2D perovskite films with different *n* values, the precursor solution compositions for various *n* values were calculated based on the chemical formula (OA)_2_(Cs_0.2_FA_0.8_)_n‐1_Pb_n_(I_0.7_Br_0.3_)_3n+1_, as shown in Table 1. After that, 1 mL of solvent was added at a DMSO:DMF volume ratio of 1:4, followed by mixing until homogeneous.

**TABLE 1:** Ingredients list for 2D perovskite precursor with different n‐values.

n‐value	OABr (mg)	CsI (mg)	FAI (mg)	PbBr_2_ (mg)	PbI_2_ (mg)	OAI (mg)
n = 1	168.12	/	/	29.36	331.92	205.73
n = 2	168.13	20.78	55.03	7.34	359.59	/
n = 3	112.08	27.71	73.37	48.93	307.34	/
n = 4	84.06	31.17	82.54	69.73	281.21	/

To fabricate PSCs, the SnO_2_ precursor was first prepared by mixing SnO_2_ colloid precursor with deionized (DI) water at a volume ratio of 1:3. PbI_2_ (253.55 mg mL^−1^), PbBr_2_ (165.15 mg ml^−1^), CsI (52 mg ml^−1^) and FAI (137.6 mg ml^−1^) were dissolved in a DMF/DMSO = 4:1 solvent for the Cs_0.2_FA_0.8_Pb(I_0.7_Br_0.3_)_3_ precursor. OABr was dissolved in IPA solvent at concentrations of 0.5, 1, 3, 6 mg/mL, etc., where the concentration unit mg/mL is defined as the mass of OABr (in mg) per volume of IPA solvent (in mL). All solutions were allowed to stand for 12 hours in N_2_‐filled glovebox at 25°C after preparation, confirming the homogeneity of the precursor solutions. The dissolution photographs of different conditions are shown in Figure S10. The spiro‐OMeTAD precursor solution was prepared by dissolving 73 mg of spiro‐OMeTAD in 1 mL CB, followed by the addition of 18 𝜇L Li‐TFSI solution (520 mg in 1 mL acetonitrile), 29 𝜇L FK209 Co(III) TFSI solution (250 mg in 1 mL acetonitrile), and 30 𝜇L TBP.

### 4.3 Film and Device Fabrication

2D perovskite layers with different n values were fabricated by spin‐coating (4000 rpm, 20 s) and annealed at 70°C for 30 min. The ITO‐coated glass substrates were cleaned through sequential sonication in DI water and ethanol (15 min each) and dried with nitrogen gas. A SnO_2_ film was obtained by spin‐coating 120 𝜇L of precursor solution onto the cleaned substrates (4000 rpm, 30 s), followed by a thermal treatment at 180°C for 20 min and a UV‐ozone treatment for 15 min before being transferred to the glovebox. Perovskite layers were formed via spin‐coating of the precursor solution using a two‐step program (1000 rpm for 10 s and 4000 rpm for 30 s). 100 𝜇L of CB was dropped on the substrates 10 s before the end of spin‐coating. The films were subsequently annealed on a hotplate at 100°C for 30 min. As for the OABr modification layer, 100 𝜇L OABr precursor was introduced to the substrates with a spin‐coating process (5000 rpm for 30 s). the substrates were then transferred to a 100°C hot plate and treated for 5 min. The perovskite films were coated with the spiro‐OMeTAD precursor by spin‐coating (4000 rpm for 30 s). The fabrication was concluded by depositing a 100 nm gold electrode on the films using thermal evaporation.

### 4.4 Characterizations

To analyze the crystal structure, XRD was performed using a Bruker D2 PHASER diffractometer with a Cu Kα source at 30 kV and 10 mA. The XPS spectra were collected on a Thermo Fisher ESCALAB 250Xi system to examine elemental composition. PL and TRPL measurements were performed by the FLS 980 fluorescence spectrometer. The PL was acquired by using a xenon lamp as the excitation source, with an excitation wavelength set at 300 nm to obtain the spectra of the perovskite films. This choice ensures that the 3D PL peak intensity remains above the noise level while extending the detection range to capture potential 2D perovskite signals. To investigate the ion migration on perovskite films, the TOF‐SIMs IONTOF M6 instrument (IONTOF GmbH) was employed. During the acquisition phase, a Bi^3+^ (30 keV) primary ion beam was used, and the ion current is 0.43 pA, while an Ar‐GCIB Ion Gun (10 kV) was used in the sputter phase. The raster size is 150 𝜇m × 150 𝜇m while the sputter area is 600 𝜇m × 600 𝜇m. The surface and cross‐sectional morphology of the films were measured by a Field Emission Scanning Electron Microscope (Regulus‐8230). The surface roughness of samples was obtained by Bruker DI Multimode 8 atomic force microscope (AFM). *J‐V* measurements were conducted using a test system comprising a standard sunlight simulator (SS‐F5‐3A) from Enlitech and a Keysight Technologies B2901A source meter. The illumination intensity was calibrated to AM 1.5G using a standard silicon reference cell (SRC‐00205). The QE‐R3018 Quantum Efficiency Test System from Enlitech was employed to get EQE data.

## Author Contributions

Y. Y., X. H. and D. Z. conceived the study and designed the experimental framework. Y. Y. and T. J. carried out the fabrication of 2D perovskite thin films. J. H. and T. J. helped the fabrication and characterization of PSCs. Y. Y. did the XRD of perovskite thin films with varied n values. Y. Y., J. H., T. J. and W. W. did the XRD, SEM, AFM, XPS, UPS characterizations of perovskite thin films. Y. Y. executed the TOF‐SIMS of perovskite thin films and *J*‐*V*, EQE, long‐term stability characterizations of the devices. Y. W. performed PL and TRPL measurements. L. W., J. Z., G. Z., M. H. and B. F. helped analyzing the data. Y. Y. and X. H. wrote the initial manuscript draft with D. Z. providing critical support in its refinement. All authors engaged in results discussions and manuscript review. X. H. and D. Z. supervised the project.

## Funding

The work was supported by the National Key R&D Program of China (Grant No. 2022YFB4200304) and the Science and Technology Project of Yibin City (2023JB001), Sichuan Province Science and Technology Support Program (2023YFG0097), Engineering Featured Team Fund of Sichuan University (2020SCUNG102).

## Conflicts of Interest

The authors declare no conflicts of interest.

## Supporting information




**Supporting File**: advs76791‐sup‐0001‐SuppMat.docx.

## Data Availability

The data that support the findings of this study are available from the corresponding author upon reasonable request.
