# Supplementary material for: Precise Management of n‐Value Distribution at the 2D/3D Interface Enabling Efficient Wide‐Bandgap Perovskite Solar Cells
Source: Adv Sci (Weinh). 2026 Aug 10;13(58):e76791. doi: 10.1002/advs.76791 (PMC13454978; doi:10.1002/advs.76791)
Supplement: Supplementary file 1 [file ADVS-13-e76791-s001.docx]

**Supporting Information**

*of*

**Precise Management of n-Value Distribution at the 2D/3D Interface Enabling Efficient Wide-Bandgap Perovskite Solar Cells**

Yajie Yang,^a^ Jiaxin Ha,^a^ Yang Zhang,^a^ Yunfan Wang,^b^ Ting Jiang,^a^ Jingquan Zhang,^ac^ Lili Wu,^ac^ Wenwu Wang,^ac^ Guanggen Zeng,^ac^ Baoyan Fan,^d^ Mohammad Abdul Halim,^e^ Xia Hao,^*ac^ Dewei Zhao^*ac^

^a^ Institute of New Energy and Low-Carbon Technology & College of Materials Science and Engineering, Sichuan University, Chengdu 610065, China

^b^ Department of Materials Science and Engineering, City University of Hong Kong, Hong Kong SAR 999077, China.

^c^ Engineering Research Center of Alternative Energy Materials & Devices, Ministry of Education, Chengdu 610065, China

^d^ College of Materials and New Energy, Chongqing University of Science and Technology, Chongqing 401331, China

^e^ Department of Materials science and Engineering, University of Rajshahi, Rajshahi 6205, Bangladesh

^*^Correspondence: Xia Hao ([*hao.xia0808@scu.edu.cn*](mailto:hao.xia0808@scu.edu.cn)), Dewei Zhao (*dewei.zhao@scu.edu.cn or dewei_zhao@hotmail.com*)

**Figure S1.** XRD patterns of (OA)_2_(Cs_0.2_FA_0.8_)_n-1_Pb_n_(I_0.7_Br_0.3_)_3n+1_ perovskites with varied n-values

**Figure S2.** XPS Pb 4f patterns of OABr treated films before and after annealing process

**Figure S3.** TOF-SIMS results of the (a) OABr-A and (b) OABr-NA films surface

**Figure S4.** Deconvolution of the 2D PL peaks for different OABr concentrations and treatment conditions. (a) OABr (1 mg/mL)-NA, (b) OABr (1 mg/mL)-A, (c) OABr (3 mg/mL)-NA, (d) OABr (3 mg/mL)-A, (e) OABr (6 mg/mL)-NA, (f) OABr (6 mg/mL)-A. The fitting parameters (peak center, FWHM, peak area, and adjusted R²) are annotated within each panel.

**Figure S5.** Steady-state PL spectra of perovskite films with 1 mg/mL OAI-treatment films before and after annealing

**Figure S6.** Cross-sectional SEM images of perovskite films: (a) control, (b) OABr-treated without annealing, and (c) OABr-treated with annealing

**Figure S7.** AFM images of (a) Control, (b) OABr-NA, (c) OABr-A films

**Figure S8.** UV-vis absorption spectra of control group and OABr-modified perovskite films before and after annealing

**Figure S9.** Comparison of Tauc plots of control group and OABr-modified perovskite films before and after annealing

**Figure S10**. Photographs of OABr/IPA solutions at various concentrations (0.5, 1, 2, 3, 6, and 10 mg/mL) after 12 h of standing at 25 °C, showing complete dissolution at all tested concentrations.

**Table S1**. Photovoltaic parameters of champion devices of OABr-A (1 mg/mL) and control group

| Group | PCE (%) | *V*_OC_ (V) | *J*_SC_ (mA/cm^2^) | FF (%) |
| --- | --- | --- | --- | --- |
| OABr-A  (1 mg/mL)-F | 19.29 | 1.24 | 19.58 | 79.55 |
| OABr-A  (1 mg/mL)-R | 19.12 | 1.24 | 19.59 | 78.85 |
| Control-F | 16.47 | 1.19 | 18.47 | 74.85 |
| Control-R | 16.00 | 1.18 | 18.38 | 73.41 |

**Table S2.** Summary of mean photovoltaic parameters for OABr-treated perovskite solar cells at different concentrations.

| Groups | | PCE(%) | *V_oc_* (V) | *J_sc_* (mA/cm^2^) | FF (%) |
| --- | --- | --- | --- | --- | --- |
| Control | 15.72 ± 0.31 | | 1.18 ± 0.0059 | 18.14 ± 0.30 | 73.22 ± 1.53 |
| OABr(0.5 mg/mL) | 16.26 ± 0.48 | | 1.21 ± 0.0103 | 18.27 ± 0.29 | 73.54 ± 1.99 |
| OABr(1mg/mL) | 18.24 ± 0.59 | | 1.23 ± 0.0062 | 19.12 ± 0.29 | 77.76 ± 1.53 |
| OABr(2mg/mL) | 17.27 ± 0.49 | | 1.22 ± 0.0064 | 18.56 ± 0.33 | 76.24 ± 0.95 |
| OABr(3mg/mL) | 16.70 ± 0.31 | | 1.22 ± 0.0032 | 18.05 ± 0.19 | 75.53 ± 1.56 |
